# Response to Comment by Faurby, Werdelin and Svenning

**DOI:** 10.1186/s13059-016-0942-z

**Published:** 2016-05-05

**Authors:** Stephen J. O’Brien, Klaus Peter Koepfli, Eduardo Eizirik, Warren Johnson, Carlos Driscoll, Agostinho Antunes, Anne Schmidt-Kuntzel, Laurie Marker, Pavel Dobrynin

**Affiliations:** Theodosius Dobzhansky Center for Genome Bioinformatics, Saint Petersburg State University, 41A Sredniy Avenue, St. Petersburg, 199004 Russia; Oceanographic Center, Nova Southeastern University Ft Lauderdale, 8000 N. Ocean Drive, Ft Lauderdale, FL 33004 USA; National Zoological Park, Smithsonian Conservation Biology Institute, Washington, DC 20008 USA; PUCRS, Faculdade de Biociencias, Laboratorio de Biologia Genomica e Molecular, Instituto Pro-Carivoros, Porto Alegre, 90619-900 Brazil; Laboratory of Neurogenetics, NIAAA, 5625 Fishers Lane, Rockville, MD 20852 USA; Department of Biology, Faculty of Sciences, University of Porto, Rua do Campo, Alegre, Porto, 4169-007 Portugal; Life Technologies Conservation Genetics Laboratory, Cheetah Conservation Fund, Otjiwarongo, 9000 Namibia; Cheetah Conservation Fund, Otjiwarongo, 9000 Namibia

Faurby, Werdelin, and Svenning raise some interesting and valid points around the interpretation of the genomics and fossil data on the history of the African cheetah, a species that has fascinated humankind for millennia. They emphasize a controversy to which we alluded in our article [1] and they offer an alternative explanation to the scenario we had suggested for the time and place of demographic events that preceded modern African cheetah populations. We fully agree that there are important areas of uncertainty in this conundrum and welcome the opportunity to reconsider and to offer our view into the natural history of *Acinonyx jubatus*. We do believe that the integration of fossil and molecular data seems a good way to get toward the truth, so we shall try to emphasize two considerations in our comments: first, the most parsimonious interpretation of data; and second, the robustness of available and relevant fossil and molecular data.

Some aspects to consider:

1. The extreme genetic depletion of modern cheetahs seems true and validated by Dobrynin et al., as has been suggested with multiple metrics across several decades [1–3]. Two possible historic events that could account for the genetic reduction are extended continental migration events and/or population bottlenecks (a.k.a. founder effect). We offered possible (but not proven) explanations based upon dating the latest founder effect at 10,000–12,000 during the late Pleistocene by multiple molecular methods, coincident with abrupt mammal extinction events in North America and Eurasia at that time. The genome-wide Dadi result suggested an earlier more ancient demographic contraction ~100,000 years ago.
  Something caused the massive diversity loss in their history. We suggested two scenarios: the earliest—a migration of North American cheetahs out of North America >100,000 years BP, and a second—when cheetahs went extinct from North America 10,000–12,000 years ago. It may be relevant that North American pumas show a near identical quantity of genomic depletion as African cheetahs, suggesting they were also extirpated form North America during the quaternary mammal extinction and repopulated from South America subsequently [3–5]. Was this an irrelevant coincidence or was a single North American or global event responsible? The answer is uncertain.
2. Please refer to [6, 7] which detail the latest relatively robust molecular phylogenies for the Felidae and a derivative study [8] that postulates parsimonious historic migration scenarios based upon phylogeny, the fossil record, present and past geographical occurrence, and geological sea levels. That phylogenetic study reported in [6] examined 18,853 bp of nuclear sequence (autosome, X and Y chromosome), which yielded 3440 parsimony informative sites, and mtDNA sequence, from all 37 Felidae species calibrated with 16 fossil dates [6].
  That molecular phylogeny (combined with paleontological, geological and present range) posited the arrival of the ancestors of three Felidae lineages (Ocelot preceding *Lynx*, and then preceding *Puma* lineage) into North America, around 8–8.5 million years ago (MYA). All three lineages have roots in North America with ocelot ancestors appearing first 8.0 MYA, *Lynx* ancestors 7.2 MYA, and Puma ancestors 6.7 MYA. Cheetah-like genera (*Acinonyx* lineage) appear first ~4.9 MYA. Support for this scenario comes from molecular evolutionary analyses that were supported by 13 cladistic synapomorphies (large insertion/deletions) [6].
  Sometime thereafter, the *Acinonyx* lineage ancestors, derived from the North American puma lineage precursors, would make their way back to Asia from North America. The fossil record puts the oldest cheetah fossils in Asia 3 million years before present (MYBP). Fossils of *Acinonyx* and cheetah-looking cats were in both in Asia and North America from 3 MYA to the late Pleistocene. So the comment in their title “There were never any true cheetahs in North America” depends on what we mean by “true cheetahs.”
3. Faurby, Werdelin, and Svenning argue cogently that North American cheetah fossils are all from the genus *Miracinonyx*, which is not closely related to modern *Acinonyx* cheetahs, which occur in Africa today and in Eurasia throughout the Pliocene and Pleistocene fossil deposits. Thus, the trivial name of North American “cheetah” is misleading (they imply we fell into that trap, although these authors themselves use the trivial name “cheetah” ten times in their note). The data supporting the distinctions of North American species in *Miracinonyx* from Eurasian *Acinonyx* are the bases of the controversy. A seminal and elegant report by Van Valkenburgh et al. [9] concluded that North American cheetah fossils (there were six specimens, one complete and five fragmented) were cladistically similar based upon ten synapomorphic morphological features that were distinct from Eurasian *Acinonyx* fossils. This study proposed the distinction of North America (*Miracinonyx*) and Eurasian (*Acinonyx*) genera in 1990. Faurby, Werdelin, and Svenning affirm this conclusion by stating “… *No fossils of Acinonyx are known from North America, despite the extensive fossil record from the continent, while no fossils of Miracinonyx are known outside North America.*”
  This declarative statement discounts a longstanding controversy that Van Valkenburgh et al. [9] acknowledge. First, there are several important adaptive morphometric characters that are shared between North America and Eurasian cheetah species including long slender limbs, small aerodynamic skulls, and reduced canines which Van Valkenburgh et al. [9] attribute to homoplasy or parallelisms. But at least three respected authors groups have disagreed [10–12] and opined that the adaptive similarities are evidence for homology of North American and African cheetahs supporting the modern cheetah origins in North America. Adams’ study titled “The cheetah-native American” [10], classifies the North American cheetah specimens as *Acinonyx* (precursors of modern *Acinonyx*) and suggests that pronouncing these important cursorial characters as convergence or parallelism “pushes the concept of parallel evolution to an unprecedented extreme”. Van Valkenburgh et al. [9] actually place the Eurasian *Acinonyx* and the North American *Miracinonyx* genera as sister taxa, emphasizing the difficulty in sorting this controversy cleanly. Further they state “… *The common ancestor (of Acinonyx and Miracinonyx) … could have been either Old or New World.*”
  Mark Springer, a noted expert of interpreting molecular and paleontological evidence across vertebrate taxa [13], states that “*… morphological studies of eutherian … relationships have failed to separate homology and homoplasy and have consistently been misled by the latter … one of the greatest challenges ahead for mammalian systematists is to tease apart homology and homoplasy in morphological characters*.” Springer’s study described explicitly how morphological inference erred in over 50 % of ordinal assignments within the mammalian radiations due to this issue [13].
4. Faurby, Werdelin, and Svenning refer to Barnet et al. [14] who presented a molecular phylogeny that uses ancient DNA to place the North American *Miracinonyx* as a sister to *Puma concolor* (and not modern *Acinonyx*), but they acknowledge the limited power of a 1302 bp mtDNA dataset. There are other details of the Barnett study that may be relevant. First, the actual nucleotide alignment is not presented which limits our ability to evaluate their conclusions or assess the extent of homoplasy. Further, when Barnet et al. performed a jack-knife analyses, excluding *H. jaguarundi* from the phylogenic analysis (*H. jaguarundi* is a sister taxon to *Puma concolor* [6–8]), statistical support for the sister relationship of *Puma-Miracinonyx* actually increased, an indication that molecular homoplasy was operative in their results [14]. Their conclusion that American cheetah *Miracinonyx* is not within the African-Asian *Acinonyx* group is tentative at best and has not been confirmed convincingly.
5. Faurby, Werdelin, and Svenning raise an important caveat concerning the confidence interval of the Dadi and PSMC and question the average generation of modern and ancient cheetahs. We do agree these aspects are not as firm as we would prefer. They hypothesize that “… *the simplest explanation of the patterns may be a gradual decline in* Acinonyx *as inferred by the PSMC algorithm, potentially with sharper declines in some periods as suggested by the DaDi analysis.*” Their points are true and we acknowledge the tentative aspects of our derived conclusions.
  In sum, we welcome the engagement of Faurby, Werdelin, and Svenning in the interpretation of the new and older data around the natural history for cheetah origins. They may be correct in their interpretation that Eurasian Cheetahs are the most recent ancestors of modern African cheetahs but their North American presence at one or several stages also seems plausible from the available data. We were not there at the time, so we cannot be sure of what the timing or influence of any possible North American cheetah migration to Asia might be, but both fossil and molecular evidence would be consistent with cheetah-like cats in both Asia and North America for some 3 MY.
  A correct explanation for African cheetah origins would seem to be one of three possibilities: (1) Eurasian origin exclusively; (2) North American origins exclusively and a migration across Asia to Africa; or (3) a combination of continental evolution on both continents, combined with periodic migration (and perhaps admixture) allowing both continental populations to contribute to modern African cheetah genetic disposition and locations. The resolution of these alternatives represents a fascinating challenge for the genome archeologists reading this exchange.
